# Genetically determined blood pressure, antihypertensive drug classes, and risk of stroke subtypes

**DOI:** 10.1212/WNL.0000000000009814

**Published:** 2020-07-28

**Authors:** Marios K. Georgakis, Dipender Gill, Alastair J.S. Webb, Evangelos Evangelou, Paul Elliott, Cathie L.M. Sudlow, Abbas Dehghan, Rainer Malik, Ioanna Tzoulaki, Martin Dichgans

**Affiliations:** From the Institute for Stroke and Dementia Research (ISD), University Hospital (M.K.G., R.M., M.D.), and Graduate School for Systemic Neurosciences (M.K.G.), Ludwig-Maximilians-Universität LMU, Munich, Germany; Department of Biostatistics and Epidemiology, School of Public Health (D.G., E.E., C.L.M.S., A.D., I.T.), UK Dementia Research Institute (P.E., A.D.), Health Data Research-UK London (P.E.), and MRC-PHE Centre for Environment, School of Public Health (I.T.), Imperial College London; Centre for Prevention of Stroke and Dementia, Department of Clinical Neurosciences (A.J.S.W.), University of Oxford, UK; Department of Hygiene and Epidemiology (E.E., I.T.), University of Ioannina Medical School, Greece; National Institute for Health Research Imperial College Biomedical Research Centre (P.E.), London; Institute for Genetics and Molecular Medicine (C.L.M.S.), University of Edinburgh, UK; Munich Cluster for Systems Neurology (SyNergy) (M.D.); and German Centre for Neurodegenerative Diseases (DZNE) (M.D.), Munich, Germany.

## Abstract

**Objective:**

We employed Mendelian randomization to explore whether the effects of blood pressure (BP) and BP-lowering through different antihypertensive drug classes on stroke risk vary by stroke etiology.

**Methods:**

We selected genetic variants associated with systolic and diastolic BP and BP-lowering variants in genes encoding antihypertensive drug targets from genome-wide association studies (GWAS) on 757,601 individuals. Applying 2-sample Mendelian randomization, we examined associations with any stroke (67,162 cases; 454,450 controls), ischemic stroke and its subtypes (large artery, cardioembolic, small vessel stroke), intracerebral hemorrhage (ICH, deep and lobar), and the related small vessel disease phenotype of white matter hyperintensities (WMH).

**Results:**

Genetic predisposition to higher systolic and diastolic BP was associated with higher risk of any stroke, ischemic stroke, and ICH. We found associations between genetically determined BP and all ischemic stroke subtypes with a higher risk of large artery and small vessel stroke compared to cardioembolic stroke, as well as associations with deep, but not lobar ICH. Genetic proxies for calcium channel blockers, but not β-blockers, were associated with lower risk of any stroke and ischemic stroke. Proxies for calcium channel blockers showed particularly strong associations with small vessel stroke and the related radiologic phenotype of WMH.

**Conclusions:**

This study supports a causal role of hypertension in all major stroke subtypes except lobar ICH. We find differences in the effects of BP and BP-lowering through antihypertensive drug classes between stroke subtypes and identify calcium channel blockade as a promising strategy for preventing manifestations of cerebral small vessel disease.

Stroke ranks among the leading causes of death and disability worldwide.^1,2^ High blood pressure (BP) is the major risk factor for both ischemic and hemorrhagic stroke, accounting for ∼50% of the population attributable risk worldwide.^3–6^ BP lowering reduces stroke risk with known differences between antihypertensive drug classes.^7,8^ Randomized controlled trials (RCTs) found calcium channel blockers (CCBs) to be superior to other drug classes, and specifically β-blockers (BB), in lowering stroke risk.^7,9,10^ However, it remains unknown whether the effects of BP or BP lowering through specific drug classes vary between stroke etiologies. In light of largely variable mechanisms between large artery stroke (LAS), cardioembolic stroke (CES), small vessel stroke (SVS), and deep and lobar intracerebral hemorrhage (ICH),^11,12^ differences seem possible and might have relevance for therapeutic decisions.

Mendelian randomization uses genetic variants as proxies for traits of interest and is by design less prone to confounding and reverse causation than observational studies.^13^ As such, Mendelian randomization has been proven valuable in exploring causality and in predicting the effects of interventions,^13–17^ as we recently showed for the effects of antihypertensive drugs on vascular outcomes.^18^ The large samples in genome-wide association studies (GWAS) further permit exploration of outcomes for which there are no adequate data from RCTs, as is the case for BP-lowering and stroke subtypes. Here, leveraging genetic data on BP^19^ and stroke,^20^ we employed Mendelian randomization to examine the effects of genetically determined BP and genetic proxies for antihypertensive drug classes on stroke subtypes, as well as on white matter hyperintensities (WMH), a radiologic manifestation of small vessel disease (SVD).

## Methods

### Standard protocol approvals, registrations, and patient consents

This study was conducted in accordance with the guidelines for Strengthening the Reporting of Observational Studies in Epidemiology– Mendelian randomization (STROBE-MR).^21^ All data were derived from studies that had already obtained ethical review board approvals.

### Genetic instrument selection

Data sources are detailed in table 1. We used summary statistics from the discovery GWAS meta-analysis of the International Consortium for Blood Pressure (ICBP) and the UK Biobank (UKB), based on 757,601 individuals of European ancestry.^19^ In the pooled sample, mean systolic BP (SBP) and diastolic BP (DBP) were 138.4 (SD 21.5) and 82.8 (SD 11.4) mm Hg, respectively. As genetic instruments for SBP and DBP, we selected single nucleotide polymorphisms (SNPs) associated with SBP or DBP at genome-wide significance level (*p* < 5 × 10^−8^) and clumped for linkage disequilibrium (LD) to r^2^ < 0.001 based on the European 1,000 Genomes panel. We estimated the proportion of variance in SBP and DBP explained by each instrument^22^ and calculated F statistics to measure instrument strength (tables e-1 and e-2, doi.org/10.5061/dryad.dfn2z34wj).^23^

**Table 1:**
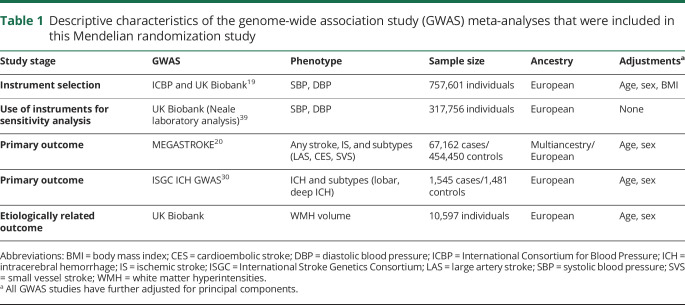
Descriptive characteristics of the genome-wide association study (GWAS) meta-analyses that were included in this Mendelian randomization study

**Figure 1:**
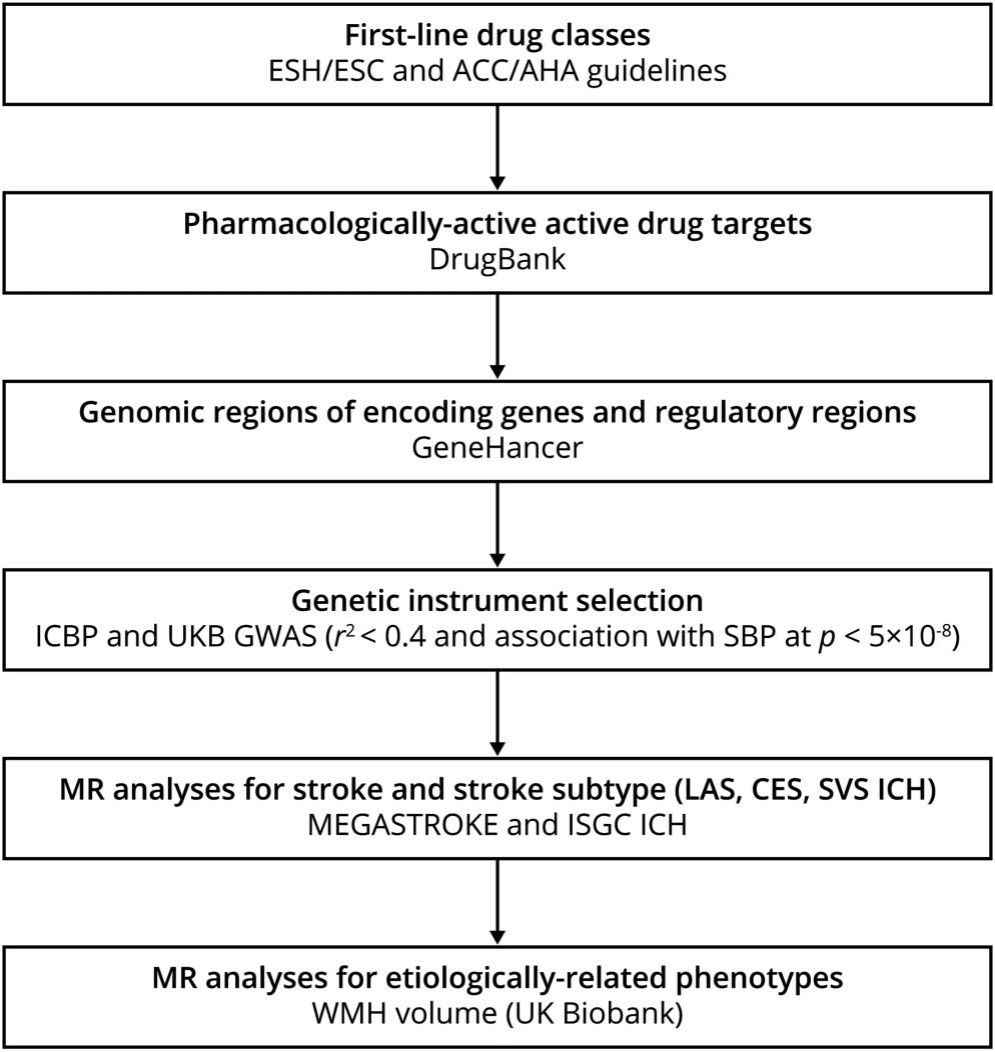
Selection strategy for genetic variants used as proxies for antihypertensive drug classes Steps for genetic instrument selection and the respective criteria and resources. ACC = American College of Cardiology; AHA = American Heart Association; CES = cardioembolic stroke; ESC = European Society of Cardiology; ESH = European Society of Hypertension; GWAS = genome-wide association studies; ICBP = International Consortium for Blood Pressure; ICH = intracerebral hemorrhage; LAS = large artery stroke; MR = Mendelain randomization; SBP = systolic blood pressure; SVS = small vessel stroke; UKB = UK Biobank; WMH = white matter hyperintensity.

We further selected genetic variants as proxies for the SBP-lowering effects of common antihypertensive drug classes (figure 1). According to our previously described strategy,^18^ we identified the genes encoding pharmacologic targets related to BP-lowering for common antihypertensive drug classes in DrugBank^24^ and screened the genomic regions corresponding to these genes and their regulatory regions (promoters and enhancers).^25^ For the main analyses, we selected SNPs associated with SBP at genome-wide significance (*p* < 5 × 10^−8^) that were at moderate to low LD (r^2^ < 0.4) according to previously described approaches,^26–28^ with sensitivity analyses using a more stringent LD threshold (r^2^ < 0.1) (table e-3, doi.org/10.5061/dryad.dfn2z34wj). The genes and the specific genomic regions screened for identification of genetic proxies for each antihypertensive drug class are detailed in table e-4 (doi.org/10.5061/dryad.dfn2z34wj).

### Primary outcomes and etiologically related phenotypes

The primary outcomes for our analyses were any stroke, ischemic stroke and its Trial of Org 10172 in Acute Stroke Treatment (TOAST)–defined subtypes (LAS, CES, SVS),^29^ or ICH and its location-specific subtypes, i.e. lobar (originating at cerebral cortex or cortical–subcortical junction) and deep (originating at thalamus, internal capsule, basal ganglia, deep periventricular white matter, cerebellum, or brainstem).^30^ Genetic association estimates for any stroke, ischemic stroke, and its subtypes were obtained from the MEGASTROKE multiethnic GWAS meta-analysis of 67,162 cases (60,341 ischemic stroke, 6,688 LAS, 9,006 CES, 11,710 SVS) and 454,450 controls.^20,31^ For ICH, we used the summary statistics from the International Stroke Genetics Consortium (ISGC) meta-analysis by Woo et al.^30^ including 1,545 cases (664 lobar, 881 deep) and 1,481 controls. In addition, we performed Mendelian randomization analyses for the radiologic phenotype of WMH volume, a manifestation of cerebral SVD etiologically related to SVS and ICH. We performed a GWAS analysis for total volume of WMH, derived from T1 and T2 fluid-attenuated inversion recovery images in the UKB data following a previously described approach,^32^ as detailed in e-Methods (doi.org/10.5061/dryad.dfn2z34wj).

### Statistical analysis

For SBP and DBP, we calculated individual Mendelian randomization estimates and standard errors from the SNP–exposure and SNP–outcome associations using the Wald estimator and the Delta method; second-order weights were used.^33^ The Mendelian randomization associations for SBP and DBP with the primary outcomes were estimated by pooling individual Mendelian randomization estimates using fixed-effects inverse variance weighted (IVW) meta-analyses.^33^ All Mendelian randomization associations between SBP, DBP, and stroke were scaled to 10 mm Hg increment in SBP and 5 mm Hg in DBP.

For the antihypertensive drug classes, including instruments at moderate to low LD (r^2^ < 0.4), we applied generalized linear regression analyses weighted for the correlation between the instruments, as previously described.^26^ This relatively lenient LD correlation threshold allows for an increase in proportion of variance explained and thus in statistical power.^26,27^ In sensitivity analyses, we restricted our instrument selection to a lower LD correlation threshold (r^2^ < 0.1) and applied fixed-effects IVW. All Mendelian randomization associations between antihypertensive drug classes and stroke were scaled to 10 mm Hg decrease in SBP.

Mendelian randomization analyses might be biased due to pleiotropic instruments. As measures of pleiotropy, we assessed heterogeneity across Mendelian randomization estimates with I^2^ and the Cochran Q test (I^2^ > 50% and *p* < 0.05 were considered statistically significant)^34^ and the intercept obtained from Mendelian randomization–Egger regression (*p* < 0.05 considered statistically significant).^35^ We further used alternative methods (weighted–median estimator,^36^ Mendelian randomization–Egger,^35^ weighted–modal estimator^37^) with relaxing assumptions regarding pleiotropic variants. The weighted median estimator requires that at least half of the information for the Mendelian randomization analysis comes from valid instruments.^36^ Mendelian randomization–Egger regression requires that the strengths of potential pleiotropic instruments are independent of their direct associations with the outcome.^35^ The weighted modal estimator provides correct estimates under the assumption that a plurality of genetic variants are valid instruments.^37^ We further tested for the presence of pleiotropic outlier variants using the Mendelian randomization pleiotropy residual sum and outlier (MR-PRESSO) test^38^ and in sensitivity IVW Mendelian randomization analyses excluded these variants.

The genetic association estimates used in the analyses for BP were corrected for antihypertensive medication use and were adjusted for body mass index,^19^ thus introducing potential bias due to medication noncompliance or collider effects, respectively. Thus we performed sensitivity analyses using unadjusted estimates for BP from a UKB GWAS (317,756 individuals).^39^ To minimize ancestral mismatch with the European population used in the BP GWAS, in sensitivity analyses we further restricted our Mendelian randomization analyses for stroke to the MEGASTROKE European subset.

Statistical significance for all analyses was set at a 2-sided *p* value <0.05. To examine whether BP differentially associated with stroke subtypes or whether there were differential effects of antihypertensive drugs on stroke risk, we compared the derived odds ratios (ORs) by computing *z* score for the differences of their natural logarithms. All statistical analyses were undertaken in R (v3.5.0; The R Foundation for Statistical Computing) using the MendelianRandomization, TwoSampleMendelian randomization, and MR-PRESSO packages.

### Data availability

This study was based on summary statistics. The GWAS data from the ICBP and UKB meta-analysis are publicly available through the GRASP repository of the National Heart, Lung, and Blood Institute (grasp.nhlbi.nih.gov/FullResults.aspx). The data from the GWAS studies for stroke and ICH are publicly available and may be accessed through the MEGASTROKE (megastroke.org/download.html) and the ISGC (cerebrovascularportal.org/informational/downloads) websites, respectively. Data from the UKB GWAS for WMH volume may be accessed through an application to UKB. The summary data for the genetic instruments used for the purposes of the current study are available in tables e-1 to e-3 (doi.org/10.5061/dryad.dfn2z34wj).

## Results

### Genetically determined BP and risk of stroke subtypes

**Figure 2:**
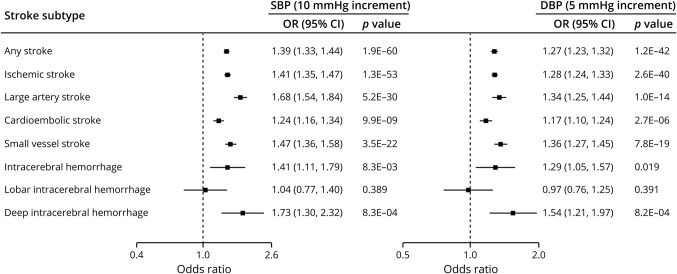
Mendelian randomization associations between genetically determined blood pressure and risk of stroke and stroke subtypes Results from the fixed-effects inverse variance weighted analysis. CI = confidence interval; DBP = diastolic blood pressure; OR = odds ratio; SBP = systolic blood pressure.

We first examined the relationship between genetically determined BP and the risk of stroke and stroke subtypes. We identified 462 genetic variants associated with SBP and 460 variants associated with DBP. F statistic was >10 for all variants, indicating low risk of weak instrument bias (tables e-1 and e-2, doi.org/10.5061/dryad.dfn2z34wj). Mendelian randomization analyses showed statistically significant associations of both SBP and DBP with risk of any stroke, ischemic stroke, and all of its major subtypes (LAS, CES, SVS), ICH, and deep ICH, but not lobar ICH (figure 2). The effects of genetically determined BP were larger for LAS and SVS compared to CES (*p* for LAS-CES comparisons of ORs = 2 × 10^−8^ for SBP and 0.004 for DBP; *p* for SVS-CES comparisons of ORs = 0.001 for SBP and 9 × 10^−4^ for DBP), and for deep compared to lobar ICH (*p* for comparisons of ORs = 0.016 for SBP and 0.009 for DBP), as depicted in figure 2.

The effect estimates remained stable in the weighted median, MR-Egger, and weighted-modal analyses, analyses excluding outliers detected with MR-PRESSO, European-restricted analyses, and analyses based on unadjusted BP estimates (table e-5, doi.org/10.5061/dryad.dfn2z34wj). Tests for heterogeneity and the MR-Egger intercepts were not significant in any of the analyses (I^2^ < 50% and *p* > 0.05, respectively), providing no evidence for pleiotropy.

### Genetic proxies for antihypertensive drugs and risk of stroke subtypes

**Figure 3:**
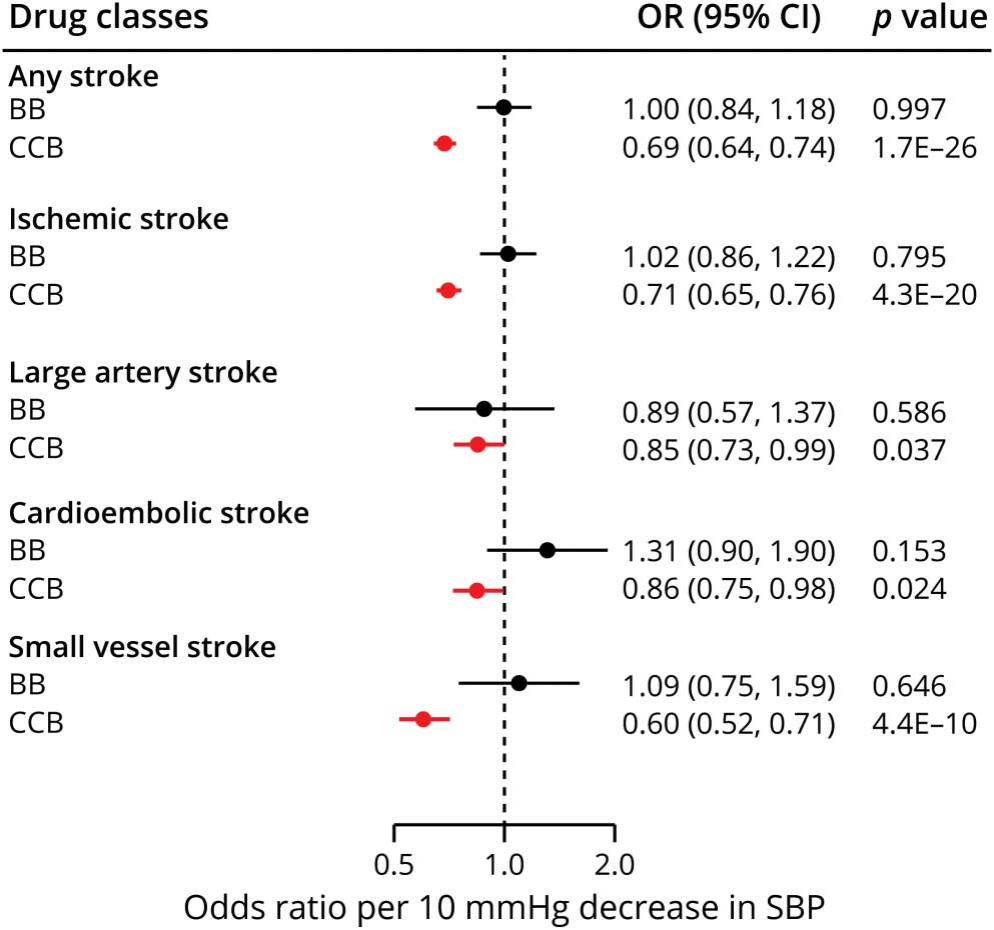
Mendelian randomization associations between genetic proxies for antihypertensive drug classes and risk of stroke and stroke subtypes Results from the Mendelian randomization analysis adjusting for correlation between variants. BB = β-blockers; CCB = calcium channel blocker; CI = confidence interval; OR = odds ratio; SBP = systolic blood pressure.

Next, we selected BP-lowering variants in genes encoding drug targets as proxies for the effects of antihypertensive drug classes, as detailed in figure 1 and as has been previously described,^18^ and examined their effects on stroke in Mendelian randomization analyses. We identified 8 proxies (variants) for BBs and 60 proxies for CCBs (table e-3, doi.org/10.5061/dryad.dfn2z34wj). We further identified a single proxy for angiotensin-converting enzyme (ACE) inhibitors, which we did not consider in the following analyses given the lack of power. A 10-mm Hg reduction in SBP through variants in genes encoding targets of CCBs, but not BBs, was associated with a significantly lower risk of any stroke and ischemic stroke (figure 3). In analyses for ischemic stroke subtypes, we found a 10-mm Hg reduction in SBP through CCB variants to be associated with significantly lower risks of LAS, CES, and SVS. The effect for SVS was stronger than that for both LAS (*p* for comparison of ORs = 0.002) and CES (*p* for comparison of ORs = 6 × 10^−4^) (figure 3). BB variants were not associated with any of the ischemic stroke subtypes. We found no significant associations for any of the drug classes for ICH and its subtypes, which is probably related to limited power (table e-6, doi.org/10.5061/dryad.dfn2z34wj).

Sensitivity analyses for BBs and CCBs restricted to the set of variants with a more stringent LD threshold (r^2^ < 0.1) showed consistent association estimates with the primary analyses for all of the examined phenotypes (table e-6, doi.org/10.5061/dryad.dfn2z34wj). For CCBs, we found no evidence for pleiotropy (heterogeneity: I^2^ < 50%; *p* of MR-Egger intercepts > 0.05). There was heterogeneity in the associations of BBs with any stroke (I^2^ = 59%), ischemic stroke (I^2^ = 67%), and SVS (I^2^ = 66%), which was however attenuated following exclusion of 2 outlier SNPs in MR-PRESSO (I^2^ = 0%, following exclusion of outlier SNPs), while the association estimates remained stable (table e-6, doi.org/10.5061/dryad.dfn2z34wj). The results remained consistent across the alternative Mendelian randomization methods (table e-6, doi.org/10.5061/dryad.dfn2z34wj).

### Genetically determined BP and WMH volume

**Figure 4:**
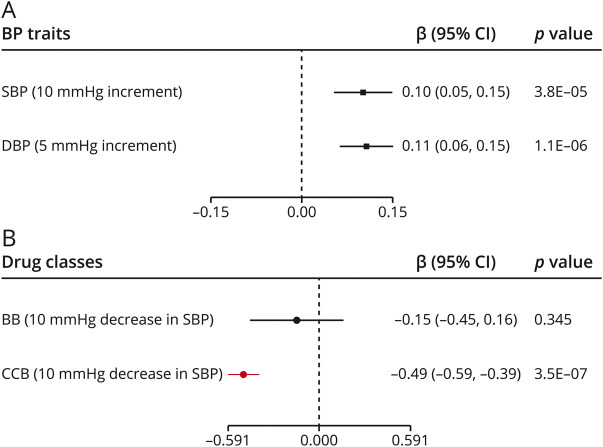
Mendelian randomization associations of (A) genetically determined blood pressure and (B) genetic proxies for antihypertensive drug classes with WMH volume Results from (A) the fixed effects inverse variance weighted analysis and (B) Mendelian randomization analysis adjusting for correlation between variants. BB = beta blockers; CCB = calcium channel blockers; CI = confidence interval; DBP = diastolic blood pressure; SBP = systolic blood pressure.

To gain additional insight into the relationship between genetically determined BP and cerebral SVD, we next calculated Mendelian randomization estimates for the associations of BP with WMH volume. We found genetically elevated SBP and DBP to be significantly associated with higher WMH volume (figure 4A). Examining the effects of genetic proxies for antihypertensive drug classes (figure 4B), we found significant associations of CCBs with lower WMH volume (β = −0.491, 95% confidence interval −0.591 to −0.391, *p* = 3.5 × 10^−7^), whereas proxies for BBs were not associated with WMH volume. The results were consistent across sensitivity analyses (table e-5, doi.org/10.5061/dryad.dfn2z34wj).

## Discussion

We investigated the relationship between the leading modifiable risk factor for stroke and etiologically defined stroke subtypes by leveraging large-scale genetic data. We found genetic predisposition to higher BP to be associated with greater risk of any stroke, ischemic stroke, each of its main subtypes, and deep but not lobar ICH. Risk was higher for LAS and SVS compared to CES. Using genetic proxies for different antihypertensive drug classes, we found BP-lowering through CCBs, but not BBs, to be associated with lower risk of stroke and ischemic stroke. CCB variants were associated with a lower risk of all major ischemic stroke subtypes, showing particularly strong effects on SVS and the related phenotype of WMH.

Our study provides evidence for a causal effect of higher BP on LAS, CES, and SVS, thus demonstrating a broad involvement of BP in the pathogenesis of ischemic stroke. Of note, however, we found the effects on stroke risk to vary depending on stroke mechanisms. Specifically, risk was more pronounced for LAS and SVS than for CES and was restricted to deep ICH. Unlike deep ICH, lobar ICH is often related to cerebral amyloid angiopathy and the absence of an association signal between BP and lobar ICH is consistent with observational data.^40,41^ As demonstrated by our drug target analyses, the effects of specific antihypertensive drug classes also differed according to stroke subtype. Collectively, these data emphasize the need to consider stroke etiologies when studying the effects of BP on stroke risk in observational and interventional studies.

Among the major findings is a benefit of BP lowering through genetic proxies for CCBs over BBs for SVS and the related phenotype of WMH. In contrast, we found no disparity in effects between genetic proxies for CCBs and BBs for LAS and CES. This suggests that CCBs may be particularly effective in preventing manifestations of cerebral SVD. The mechanisms underlying this observation are unknown but may include direct effects of CCBs on cerebral microvessels or systemic effects, for instance, from the established influence of CCBs on BP variability.^9,10,42^

Patients with cerebral SVD mark a population at increased risk for stroke, dementia, and death.^43^ SVD manifestations are highly prevalent in the aging population, with figures reaching up to 90% in patients aged 65 years and above.^44^ Yet there have been no informative trials on specific antihypertensive agents for the prevention of SVS, WMH, or other manifestations of SVD.^45–47^ Our Mendelian randomization results suggest that BP lowering with CCBs should be tested in clinical trials for prevention of SVS and other outcomes related to SVD.

The consistency of our results for stroke obtained from genetic proxies for different drug classes with those from previous RCTs^7,9,10^ is worth noting and lends confidence to our findings on etiologic stroke subtypes for which no data from RCTs exist. The disparity in treatment effects between CCBs and BBs on stroke risk has been related to the opposite actions of these drugs on BP variability; CCBs decrease whereas BBs increase BP variability.^9,10^ However, whether the effects of BP variability on stroke risk vary by stroke etiology is unresolved and deserves further investigation.

Our study has several methodologic strengths. We used large datasets offering sufficient statistical power for most analyses and applied multiple methods to exclude pleiotropic effects and other biases. We also examined phenotypes etiologically related to stroke subtypes and performed mediation analyses that allowed inferences on mechanistic aspects regarding the association of BP with stroke. Finally, we used genetic proxies for antihypertensive drug classes that have been validated previously and have shown comparable effects to those derived from RCTs.^18^

Our study also has limitations. First, Mendelian randomization examines the lifetime effects of genetically determined BP, which might differ from the effect of a clinical intervention for BP lowering. Second, based on our selection criteria, we identified only a single genetic proxy for ACE inhibitors that did not offer sufficient statistical power to perform meaningful analyses. Future studies encompassing larger GWAS datasets for BP might identify such variants and might thus offer deeper insights into differential effects between different classes of BP-lowering agents including ACE inhibitors, angiotensin-receptor blockers, and thiazide diuretics on stroke and stroke subtypes. Third, by design, we could not examine nonlinear associations between BP and stroke risk.^48^ However, current evidence suggests that the association of midlife SBP and DBP with stroke seems to follow a linear pattern.^49^ Fourth, our results apply stroke incidence and not stroke recurrence. While we found high BP to not be associated with risk of lobar ICH, hypertension has been shown in observational studies to increase the risk for both deep and lobar ICH recurrence,^50^ which could not be examined in the context of the current study. Fifth, the small sample size for the ICH GWAS did not offer sufficient power to examine the effects of antihypertensive drug classes on any, lobar, and deep ICH. Sixth, our GWAS data for BP were restricted to individuals of European ancestry, which could limit generalizability of our findings to this population. This might specifically apply for ICH^30^ given the evidence from observational studies for differential associations of BP with lobar ICH depending on ethnicity.^51^ Furthermore, there is evidence for differential responses to antihypertensive drug classes by ethnicity, which could not be examined in the current study.^52^ The availability of large-scale GWAS data from more diverse populations with higher representation of non-European ethnicities will enable future Mendelian randomization studies to explore potential ethnic disparities in more detail. Finally, it was not possible to disentangle the effects of dihydropyridine and nondihydropyridine CCBs with Mendelian randomization, because the differences in the subunits of the voltage-gated calcium channels that are the targets of these drug subclasses in the vessels and the heart, respectively, are encoded by the same genes but are the result of alternative splicing.^53^

We provide evidence for a causal association of higher BP with risk of any stroke and all stroke subtypes except lobar ICH, with a higher risk of large artery stroke and SVS compared to cardioembolic stroke. Our findings support CCBs, but not BBs, to lower ischemic stroke risk. Genetic proxies for the effects of CCBs showed particularly strong associations with SVS and WMH, highlighting calcium channel blockade as a promising strategy for the prevention of cerebral SVD.

## Glossary

ACE: angiotensin-converting enzyme
BB: β-blockers
BP: blood pressure
CCB: calcium channel blocker
CES: cardioembolic stroke
DBP: diastolic blood pressure
GWAS: genome-wide association studies
ICBP: International Consortium for Blood Pressure
ICH: intracerebral hemorrhage
ISGC: International Stroke Genetics Consortium
IVW: inverse variance weight
LAS: large artery stroke
LD: linkage disequilibrium
MR-PRESSO: Mendelian randomization pleiotropy residual sum and outlier
OR: odds ratio
RCT: randomized controlled trial
SBP: systolic blood pressure
SNP: single nucleotide polymorphism
SVD: small vessel disease
SVS: small vessel stroke
UKB: UK Biobank
WMH: white matter hyperintensity
